# The Story of the Insane from Year to Year

**Published:** 1893-09-30

**Authors:** 


### THE STORY OF THE INSANE FROM YEAR TO YEAR.

(SIXTH SERIES.)

### YORK LUNATIC HOSPITAL.

In this asylum the numbers increased from 128 to 136, the total accommodation being for 180. There were 47 admissions, 19 recoveries, and 13 deaths. Intemperance is the assigned cause in eight of the admissions, and hereditary tendency in seven. The recoveries stand at 41·73 per cent. of the admissions and the deaths at 7·23 on the total number resident. Dr. Hithcock gives a resumé of the history of the hospital for the ten years during which it has been under his superintendence, and he shows that his percentage of recoveries has been 55·16, and the average in all the registered hospitals only 46·58; while in the county and borough asylums it was a fraction under 40. While fully admitting that treatment, using the word in its widest sense, will have an important effect upon the recoveries, it cannot be denied that the nature of the admissions will have a still more important effect, and as no selection can be made in public asylums it follows that their recovery rate must necessarily be lower than in the lunatic hospitals where selection is all but invariable. If county asylums were to exclude idiots, epileptics, and paralytics their recovery rate would be very different. Dr. Hitchcock is down on sedatives,

"and he believes that their disuse contributed to his high recovery rate." In acute cases the question must always be "Will the sedative in this particular case be more hurtful to the patient than the excitement and want of sleep?" And according to the answer so ought the practice to be. We do not notice the reports of the Commissioners in Lunacy nor any table showing the cost per head per week, nor even the weekly payments made by patients. We find on the last page of the report the following paragraph relating to these payments, but not much information can be extracted from it: "Patients are admitted at two guineas a week, but the committee reserve the power to increase or reduce at their discretion the amount of such weekly payments to meet the exigencies of the case. Patients paying higher rates can have private apartments and special attendants, carriages, &c., while the income of Lupton's Charitable Fund, amounting to about £400 a year, is available in aid of payments for poorer patients on application being made to the Committee."

### MAVISBANK ASYLUM, NEAR EDINBURGH.

This is a hospital for the insane for the higher and middle classes, and it is managed by a small board of directors, who placed it under the able superintendence of Dr. Keay. On January 1st it contained 49 patients, and at the close of the year 48 were in residence. Forty-three patients were admitted during the year—38 were discharged and 6 died. The percentage of recoveries stands at 51 per cent. on the admissions. The rate of payment varies from £80 to £400 a year according to the accommodation required.

### GLOUCESTER COUNTY ASYLUMS.

An increase from 1,035 to 1,037 is to be noted during the year; but this is owing to out-county patients, for it is stated that the Gloucestershire patients have decreased by four during the year. There were 267 admissions, 103 recoveries, and 116 deaths. Hereditary influence played some part in the causation of 35 of the admissions, and drink is the assigned cause in 12. Thirty-seven of the deaths were owing to cerebral or spinal disease, and 14 to consumption, and the latter was present in 6 of the other deaths. The percentage of recoveries stands at 42·92 on the admissions, and the deaths at 11·47 on the average number resident. Both the old and new asylums are still kept going under one management, and Mr. Craddock wisely advises the committee to sell the older one and complete the new so that all the Gloucestershire patients might be under one roof. It is manifestly difficult even for such an energetic man as Mr. Craddock to keep both places thoroughly in hand, and we do not wonder at his saying he feels it impossible to give that close personal supervision to two large places so far apart as they demand. Last year we had occasion to notice that the committee had decided not to admit private patients owing to the trouble and complications caused by that unfortunate Lunacy Act. We are pleased to see that they have rescinded this resolution, and the old state of things prevails. Why cannot the committees of our County Asylums unite and agree upon some action whereby the great bulk of that Lunacy Act might somehow or other be swept from the Statute Book? Mr. Craddock has ere now written rather hard things about the Commissioners, but we admire his fairness and we find him praising those who visited his asylums during 1892, stating the pleasure he had in discussing asylum matters with them, and remarking that it is more satisfactory to deal with persons who give others credit of an honest desire to do their best for their patients. We join with Mr. Craddock in hoping the Commissioners will recognise there are two sides to every question, and we further hope they will lay to heart the quotation from the Book of Job with which he favours them. The visitors say that "under the management of Mr. Craddock the asylums have been maintained in good order, and to the entire satisfaction of the committee." The Commissioners say, "Apart from the matters specially mentioned we may report that we found both asylums in good order, and that the case books and pathological records are well kept." The weekly rate of maintenance is 8s. 1d.

### PORTSMOUTH BOROUGH ASYLUM.

On December 31st, 1892, this asylum had 540 patients in residence, being one fewer than on January 1st. One hundred and ten patients were admitted; 51 were discharged recovered, and 32 died. Put into percentages these numbers show that the recoveries stand at 48·1, and the deaths at 5·9. Dr. Bland congratulates himself and the committee on the good work the asylum is doing as evidenced by the high recovery rate, and possibly it is a source of congratulation when viewed from one standpoint; but if Dr. Bland and his committee will take a peep into the next generation, and see how this high recovery rate will increase the insanity of the future, they might wish they had rested satisfied with 40 per cent., or even less. Drink caused the insanity in 14 of the admissions, and hereditary influence in 25. The committee express their satisfaction at the able management of the asylum by Dr. Bland. The Commissioners think the wages given to the attendants and nurses are too low, and the Commissioners are right. £21 a year rising to £28 for men, and £16 rising to £22 for women is hardly enough to ensure good material. The weekly rate of maintenance is nearly 9s. 10d.

### ASYLUMS FOR THE LEEWARD ISLANDS.

The Rat Island Asylum contained seventy patients at the close of the year. There were 49 admissions, 11 recoveries, 17 discharged relieved, and 29 died. A coroner's inquest was held in every case of death, but no post-mortem examinations were made. The proportion of attendants to patients is high, being one to five, which is at least twice as many as in our English asylumns. The cubic space allowed per patient is in dormitories 708 feet, and in day-rooms 1,494 feet, the latter being more than three times as great as the allowance in England. The closets are all on the dry earth system. The asylum is supplied by rain water, and the tanks contain a supply practically unlimited. It is stated that 16 patients were "very frequently" in seclusion; 40 were "very frequently" restrained by attendants; and 10 were "very frequently" restrained by mechanical means. Here is a large amount of restraint and seclusion for so small an asylum, and it surely needs looking into by the authorities. We notice that the Governor of the island visited the asylum twice during the year, the Colonial Secretary seven times, and the committee five times. There were no injuries to patients reported, nor was there any cruelty on the part of attendants. We read that patients are allowed to go beyond the grounds and to bathe in the sea. We should like to know how many enjoy this privilege, and if the number be considerable, how can it be reconciled with the practice of so much seclusion and restraint? It will generally be found that the number of patients employed will be small in proportion as the restraint is large, and we are not surprised to learn that only 24 find employment in household work and in drawing water. There would seem to be no other occupation for them. The Ridge Asylum contains only 35 patients. Eleven patients were admitted and 9 were discharged recovered during the year, this being the highest percentage of recoveries to admission we have ever had to notice. No restraint was employed, and seclusion was used once. We find that 7 men and 5 women were employed on the farm, 1 man makes baskets, and 8 men and 9 women were engaged in household work. Like the recoveries, the percentage of patients employed is the highest we have yet met. These points come out in sharp contrast with the Rat Island Asylum, and we should like an explanation of them. The diet in both asylums is alike and bears a certain resemblance to that of our county asylums except that fish enters more largely into it, and each patient has in the early morning one pint of sweetened water.

